# Role of ACSL4 in the chemical-induced cell death in human proximal tubule epithelial HK-2 cells

**DOI:** 10.1042/BSR20212433

**Published:** 2022-02-09

**Authors:** Hiroshi Kuwata, Yuki Tomitsuka, Emiko Yoda, Shuntaro Hara

**Affiliations:** Division of Health Chemistry, Department of Healthcare and Regulatory Sciences, School of Pharmacy, Showa University, 1-5-8 Hatanodai, Shinagawa-ku, Tokyo 142-8555, Japan

**Keywords:** ACSL4, Acyl-CoA synthetase, phospholipids, polyunsaturated fatty acids

## Abstract

Acyl-CoA synthetase long-chain family member 4 (ACSL4) activates polyunsaturated fatty acids (PUFAs) to produce PUFA-derived acyl-CoAs, which are utilised for the synthesis of various biological components, including phospholipids (PLs). Although the roles of ACSL4 in non-apoptotic programmed cell death ferroptosis are well-characterised, its role in the other types of cell death is not fully understood. In the present study, we investigated the effects of ACSL4 knockdown on the levels of acyl-CoA, PL, and ferroptosis in the human normal kidney proximal tubule epithelial (HK-2) cells. Liquid chromatography–tandem mass spectrometry (LC-MS/MS) analyses revealed that the knockdown of ACSL4 markedly reduced the levels of PUFA-derived acyl-CoA, but not those of other acyl-CoAs. In contrast with acyl-CoA levels, the docosahexaenoic acid (DHA)-containing PL levels were preferentially decreased in the ACSL4-knockdown cells compared with the control cells. Cell death induced by the ferroptosis inducers RSL3 and FIN56 was significantly suppressed by treatment with ferrostatin-1 or ACSL4 knockdown, and, unexpectedly, upon treating with a necroptosis inhibitor. In contrast, ACSL4 knockdown failed to suppress the other oxidative stress-induced cell deaths initiated by cadmium chloride and sodium arsenite. In conclusion, ACSL4 is involved in the biosynthesis of DHA-containing PLs in HK-2 cells and is specifically involved in the cell death induced by ferroptosis inducers.

## Introduction

Accumulating evidence has revealed that the dietary intake of polyunsaturated fatty acids (PUFAs)^2^ may play positive and/or negative roles in human disease and health, including cardiovascular and kidney diseases [1,2]; however, the underlying biological mechanisms are still not completely understood. In general, fatty acid activation is a critical step in the biological utilisation of fatty acids, and the reactions are mediated by acyl-CoA synthetases (ACSs) [3]. The ACS family enzymes are divided into five subfamilies based on the acyl chain length of their substrates [4]. Among the ACS subfamilies, acyl-CoA synthetase long-chain family members (ACSL) activate fatty acids with chain lengths of 16–22 carbon atoms, and five ACSL isozymes have been identified in mammals. Because the tissue distribution and substrate selectivity of ACSL isozymes are different among ACSL isozymes, each ACSL isozyme is thought to have unique functions.

ACSL4 was first identified as an ACSL isozyme with arachidonic acid (AA) and eicosapentaenoic acid as a substrate and is expressed in a wide range of tissues [5]. Recent studies have revealed that not only the biosynthesis of AA- and/or eicosapentaenoic acid-derived acyl-CoA, but also those of several PUFA-derived acyl-CoAs were significantly decreased in cells obtained from *Acsl4*-deficient mice compared with those in wildtype mice [6,7]. Consistent with these observations, genetic deletion of Acsl4 significantly decreased in the levels of PUFA-containing phospholipids (PLs) in several cells, such as adipocytes [6], macrophages [7], and fibroblasts [8]. Thus, ACSL4 is critical for the maintenance of PUFA-containing PLs and is likely to contribute to various events involved in PUFA metabolism. More recently, it has been shown that oxidised PLs are a key factor in regulating the non-apoptotic form of programmed cell death called ferroptosis [9]. Thereafter, PUFA-containing PLs, such as AA- or adrenic acid-containing phosphatidylethanolamines (PEs) produced via the ACSL4 pathway, are potential targets of oxidative stress, and their peroxidation products have been identified as key factors for the execution of ferroptosis [8,10]. Drugs and therapies that induce ferroptosis are anticipated as alternative treatments for apoptosis-resistant cancers [11–13]. However, ferroptosis is also known to be involved in drug-induced adverse reactions [14]. Thus, it is important to understand the mechanisms and functions of ferroptosis under various pathological and physiological conditions. Furthermore, ferroptosis contributes to various diseases such as renal diseases [15]. Up-regulation of ACSL4 expression was found in patients with acute kidney tubular injury, and ferroptosis plays an important role in the progression of renal diseases [16,17]. However, the role of ACSL4 in the ferroptotic cell death of the renal cells is not fully understood. To address these issues, we investigated the effects of ACSL4 knockdown on ferroptosis of renal cells by using the human normal kidney proximal tubule epithelial (HK-2) cells.

## Materials and methods

### Reagents

Lipofectamine RNAiMAX reagent and Opti-MEM medium were obtained from Invitrogen (Carlsbad, CA, U.S.A.). RSL3 was purchased from Selleckchem (Houston, TX, U.S.A.). The mouse monoclonal antibody against β-actin, the rabbit polyclonal antibody against ACSL6 (SAB4500971), NaAsO_2_, heptadecanoyl CoA (17:0-CoA), and 2′,7′-dichlorodihydrofluorescein diacetate (DCFH-DA), 1,2-dimyristoyl-*sn*-glycero-3-phosphocholine (14:0/14:0 phosphatidylcholine (PC)), 1,2-dimyristoyl-*sn*-glycero-3-phosphoethanolamine (14:0/14:0 PE), and 1,2-dimyristoyl-*sn*-glycero-3-phospho-(1′-rac-glycerol) (14:0/14:0 phosphatidylglycerol), were obtained from Sigma–Aldrich (St. Louis, MO, U.S.A.). The rabbit monoclonal antibody against ACSL4 (FACL4: ab155282) and the rabbit polyclonal antibody against ACSL5 (ab104892) were acquired from Abcam (Cambridge, MA, U.S.A.). Ferrostatin-1 was obtained from Cayman Chemicals (Ann Arbor, MI, U.S.A.). Necrostatin-1 was acquired from ChemScene LLC (Monmouth Junction, NJ, U.S.A.). CdCl_2_ was purchased from Wako Chemicals (Osaka, Japan). The rabbit polyclonal antibody against ACSL1 (H-65), the mouse monoclonal antibody against ACSL3 (H-9), and DPQ were purchased from Santa Cruz Biotechnology (Santa Cruz, CA, U.S.A.). Z-VAD-FMK (z-VAD) was obtained from the Peptide Institute, Inc. (Osaka, Japan).

### Cell culture

The HK-2 cells (human normal kidney proximal tubule epithelial cells) were obtained from the American Type Culture Collection (ATCC; Manassas, VA, U.S.A.). The HK-2 cells were cultured in Dulbecco’s modified Eagle’s medium containing 10% (v/v) foetal calf serum (FCS), penicillin/streptomycin (100 units/ml and 100 µg/ml, respectively), and 2 mM glutamine (Life Technologies, Paisley, U.K.) under a humidified atmosphere containing 5% CO_2_.

### Transfection of small interfering RNA

HK-2 cells (5 × 10^4^ cells/ml) were seeded in 12-well plates (for immunoblotting, measurement of acyl-CoA, and PLs) or 96-well plates (for cell viability analysis) and cultured for 24 h before transfection. The small interfering RNA (siRNA) against ACSL4 (siRNA ID number SASI_Hs01_00114667; Sigma–Aldrich) or a negative control siRNA (Sigma–Aldrich) was transfected into the HK-2 cells using Lipofectamine RNAiMAX according to our protocol [18]. The siRNA transfection was carried out at a final concentration of 10 nM.

### Immunoblotting

A 10-µl aliquot of cell lysate was subjected to SDS/PAGE using a 10% (w/v) gel under reducing conditions. The separated proteins were electroblotted on to nitrocellulose membranes (GE Healthcare Bioscience, Piscataway, NJ, U.S.A.) with a bath-type blotter. After blocking for 1 h with 5% (w/v) skimmed milk in phosphate-buffered saline (PBS) containing 0.05% Tween 20 (TPBS), the membranes were probed for 1 h with the respective antibodies (1:1000 for ACSL1, ACSL3, ACSL5, and ACSL6, 1:5000 for ACSL4, and 1:10000 for β-actin), followed by incubation with horseradish peroxidase-conjugated anti-rabbit (1:3000 for ACSL1, ACSL4, ACSL5, and ACSL6) and anti-mouse (1:3000 for ACSL3 and β-actin) IgG. After washing, the membranes were visualised using Western Lightning Chemiluminescence Reagent Plus (PerkinElmer Life Sciences, Wellesley, MA, U.S.A.).

### Real-time PCR

RNA extraction, cDNA synthesis, and real-time PCR were carried out according to our standard protocols, as previously described [18]. The following primers were used: ACSL1, sense 5′-GCT CTC GGA AAC CAG ACC AA-3′ and antisense 5′-AAG CCC TTC TGG ATC AGT GC-3′; ACSL3, sense 5′-TGT TGA TGG AAA GCC ACC GA-3′ and antisense 5′-GTT TTC CAT GCT GGC CTT GG-3′; ACSL4, sense 5′-GGA ATG ACA GGC CAG TGT GA-3′ and antisense 5′-TAG CAC ATG AGC CAA AGG CA-3′; ACSL5, sense 5′-GCC CCC ATT CAC TAG AAG CA-3′ and antisense 5′-TCA GGA TGC AGA TCA ACG CC-3′; ACSL6, sense 5′-GAC CTT CTT CCT CGT GTC GG-3′ and antisense 5′-GTC ACC TAG CTC AGG CAG TC-3′; 18S ribosomal RNA, sense 5′-CGA ACG TCT GCC CTA TCA ACT T-3′ and antisense 5′-ACC CGT GGT CAC CAT GGT A-3′. 18S ribosomal RNA was used as internal housekeeping gene.

### Extraction and liquid chromatography–tandem mass spectrometry analysis of acyl-CoAs

ACSL4 knockdown or control HK-2 cells (4 × 10^5^ cells) were resuspended in 500 µl of methanol containing 1 nM 17:0-CoA, vortexed vigorously, and centrifuged at 15000 rpm for 10 min at 4°C. The supernatants were dried using a rotary evaporator to remove the solvent, and 50 µl of methanol was used to dissolve the acyl-CoAs. After centrifugation at 15000 rpm for 10 min at 4°C, the supernatants were analysed by liquid chromatography–tandem mass spectrometry (LC-MS/MS) as described previously [7,19].

### LC-MS/MS analysis of PLs

For the analysis of PLs, total lipids were extracted from the ACSL4-knockdown and control HK-2 cells using methods established by Bligh and Dyer [20]. A mixture of PL internal standards (PC (14:0/14:0), 10 pmol; PE (14:0/14:0), 10 pmol; and phosphatidylglycerol (14:0/14:0), 1 pmol) was added to each sample during the first lipid extraction step to quantify the analytes. The organic phase was recovered and evaporated to dryness using a rotary evaporator. Samples were resuspended in 200 µl of solvent A of normal phase LC (2-propanol/tert-butyl methyl ester/ammonium formate = 34/17/5) and injected into an LC-MS/MS system. All mass spectrometric analyses were performed using a Prominence HPLC system (Shimadzu, Kyoto, Japan) equipped with a linear ion trap quadrupole mass spectrometer (QTRAP5500, Sciex, Framingham, MA, U.S.A.). Separation of the PL class by normal-phase LC was performed as previously reported by Kim and Hoppel [21], except for the separation column (Inertsil SIL-100A column (2.1 × 150 mm, GL Science, Tokyo, Japan)). The discrimination of PL species with different fatty acid side chains was performed using the negative-ion multiple-reaction monitoring (MRM) approach. In the MRM transitions (Q1 → Q3), the [M + HCOO]^−^ precursor ions were used as Q1 for the detection of PC species, whereas the [M–H]^−^ precursor ions were used as Q1 for the detection of PE, phosphatidylinositol (PI), phosphatidylserine (PS), and phosphatidylglycerol species. The *m/z* of long-chain fatty acids ([M–H]^−^ ions) was set in Q3. The MRM transitions required for PL quantification are presented in Supplementary Table S1.

### Cell viability assay

Cell viability was assessed using Cell Counting Kit-8 (Dojindo, Kumamoto, Japan). HK-2 cells (5 × 10^3^ cells/well) were seeded in a 96-well plate overnight, and then, the medium was replaced with 100 µl of DMEM supplemented with 0.5% (v/v) FCS in the presence or absence of RSL-3, cadmium chloride (CdCl_2_), or sodium arsenite (NaAsO_2_). After culturing for 6, 24, and 48 h, the Cell Counting Kit-8 solution was added to each well and incubated for an additional 3 h at 37°C. The absorbance of each well was measured using an iMark™ microplate reader (Bio-Rad, Hercules, CA, U.S.A.) at a wavelength of 450 nm. To investigate the effects of various inhibitors, the cells were pre-treated for 1 h with or without various inhibitors (1 µM Ferrostatin-1 (Fer-1), 20 µM z-VAD, 10 µM DPQ, or 10 µM Necrostatin-1 (Nec-1)) before the addition of various chemicals. To assess the cell viability of HK-2 cells against FIN56, cell viability was evaluated in subsequent experiments 48 h after FIN56 treatment.

### Detection of reactive oxygen species

The intracellular reactive oxygen species (ROS) levels in HK-2 cells were measured using a DCFH-DA assay. HK-2 cells were cultured in 35-mm glass-bottom dishes (Matsunami Glass Ind., Ltd., Osaka, Japan) at 5 × 10^4^ cells/ml for 24 h. After cultivation, the cells were incubated with the indicated concentrations of CdCl_2_, NaAsO_2_, or RSL3 in HBSS (−) for 6 h. After washing once with HBSS (−), the cells were incubated with 20 µM DCFH-DA fluorescence probe and 5 µg/ml Hoechst 33258 in HBSS (−) for 20 min at 37°C. Subsequently, the images of ROS-positive cells in each dish (at least four independent fields) were captured using a fluorescence microscope (BZ-X810: Keyence Corporation, Osaka, Japan), and the percentage of ROS-positive cells (ROS-positive cells/Hoechst-positive cells × 100) in random areas were analysed by a hybrid cell count software (Keyence).

### Statistical analysis

GraphPad Prism software (Version 8.4.3; San Diego, CA, U.S.A.) was used for the analysis of the results. Comparisons between the two groups were made with an unpaired Student’s *t* test. Differences were considered significant at values of *P*<0.05.

## Results

### Comparison of the acyl-CoA and PL levels between the ACSL4 knockdown and control HK-2 cells

**Figure 1:**
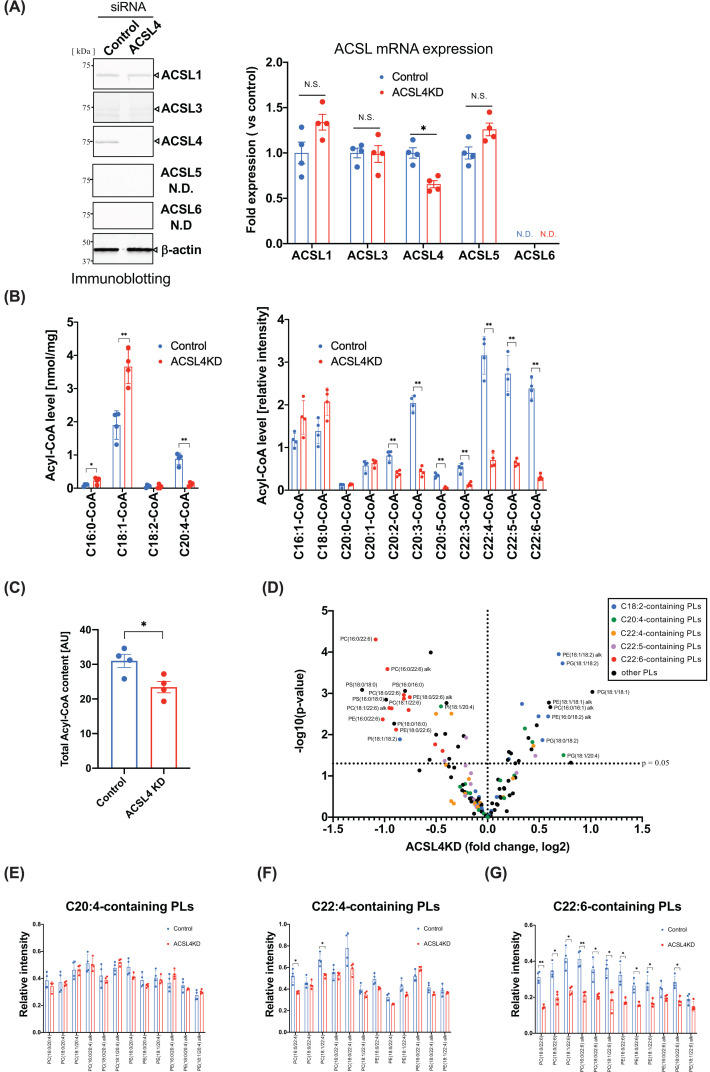
Knockdown of ACSL4 in HK-2 cells The HK-2 cells were transiently transfected with ACSL4 or control siRNA (10 nM). Two days after transfection, the HK-2 cells were subjected to immunoblotting (**A**), or LC-MS/MS analysis for the quantification of fatty acyl-CoA (**B,C**) and PLs (**D–G**). (A) Immunoblot (*left panel*) and real-time PCR (*right panel*) of ACSL enzymes in the ACSL4 knockdown or control cells. Equal loading of samples in each lane was immunoblotted with anti-β-actin antibody. Data represent the mean ± SD. *, *P*<0.05; N.S., not significant; N.D., not detected. (B) Long-chain fatty acyl-CoA levels of ACSL4 knockdown HK-2 cells compared with those of the control cells. (C) The area under the curves of acyl-CoA fractions of control and ACSL4 knockdown HK-2 cells were quantitated using LC-MS/MS. Data represent the mean ± SD. *, *P*<0.05; **, *P*<0.01. (D) Data are represented as volcano plot coloured by the fatty acid component of PLs. Significant differential abundance for PL species between control and ACSL4 knockdown HK-2 cells was assigned to *P*<0.05. (E–G) The levels of PEs and PCs with AA (C20:4) (E), adrenic acid (C22:4) (F), or docosahexaenoic acid (22:6) (G) in ACSL4 knockdown (red bars) and control (blue bars) HK-2 cells. Data represent the mean ± SD. *, *P*<0.05; **, *P*<0.01.

To evaluate the effects of ACSL4 knockdown on cell death induced by chemicals in HK-2 cells (human kidney proximal tubule epithelial cells), we investigated the levels of long-chain acyl-CoA and PL in ACSL4 knockdown cells compared with the control cells. We first attempted to detect ACSL enzymes in HK-2 cells using immunoblotting and real-time PCR. As shown in Figure 1A, ACSL1 and ACSL4 were the main ACSL enzymes expressed in HK-2 cells as judged by immunoblotting. The protein expression of ACSL3 was also slightly detected, although those of ACSL5 and ACSL6 were not detected in HK-2 cells. Transfection of ACSL4 siRNA significantly attenuated the ACSL4 protein and mRNA expression compared with that of the control siRNA without any effects on other ACSL enzyme protein and mRNA expressions (Figure 1A and Supplementary Figure S1). Next, we assessed the effects of ACSL4 knockdown on the levels of long-chain acyl-CoAs in HK-2 cells using LC-MS/MS. As shown in Figure 1B, consistent with previous reports [6,7], knockdown of ACSL4 markedly reduced the levels of long-chain acyl-CoA derived from fatty acids (carbon 20 and 22) with more than two double bonds, such as AA (C20:4), and adrenic acid (C22:4). These results show the possibility that ACSL4 participates in the biosynthesis of long-chain PUFA-derived acyl-CoA in HK-2 cells, which is consistent with substrate selectivity of recombinant ACSL4 enzyme [7]. To assess the contribution of ACSL4 on the biosynthesis of total levels of long-chain acyl-CoA content, we compared the total levels of acyl-CoA species in control and ACSL4 knockdown HK-2 cells using LC-MS/MS. As shown in Figure 1C, the net amounts of long-chain acyl-CoA levels in ACSL4 knockdown HK-2 cells were reduced by 25% in comparison with those of control cells; this shows that ACSL4 is one of major ACSL enzymes producing the long-chain acyl-CoA in these cells. Contrary to the levels of PUFA-derived acyl-CoA, those of some saturated or monounsaturated fatty acid-derived acyl-CoA were increased in ACSL4 knockdown cells.

Next, we examined whether the suppression of the ACSL4 expression altered the fatty acid composition of PLs in these cells. While knockdown of ACSL4 significantly decreased the levels of PC and PE with docosahexaenoic acid (DHA) (C22:6), the levels of PLs with linoleic acid (C18:2) or oleic acid (C18:1) were higher in ACSL4 knockdown cells than in control cells (Figure 1D–G). These results suggest that while knockdown of ACSL4 decreased the levels of several PUFA-derived acyl-CoA, its effects on PLs were restricted to C22:6-containing PLs. Further, the levels of PC and PE with C20:4, a well-known substrate of ACSL4, were not decreased in ACSL4 knockdown cells compared with those in control cells even though the level of C20:4-CoA was markedly decreased (Figure 1D,E).

### Effects of ACSL4 knockdown on RSL3-induced ferroptotic cell death in HK-2 cells

**Figure 2:**
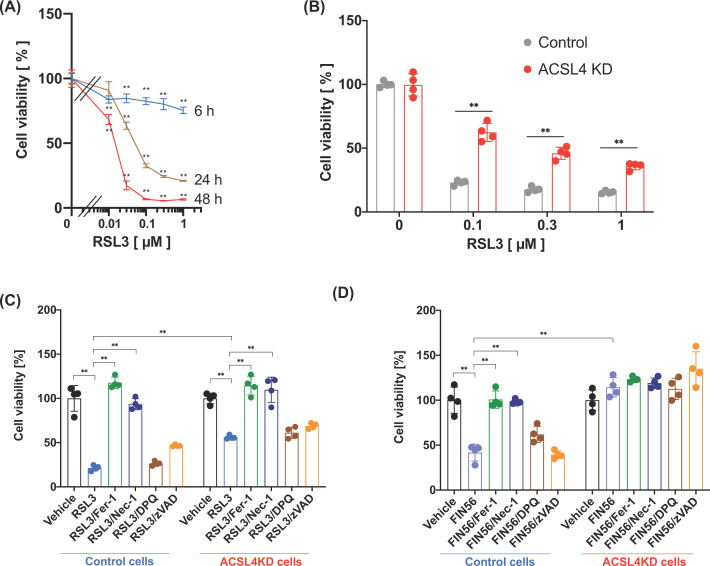
Effects of RSL3 on HK-2 cell survival The HK-2 cells grown in a 96-well plate were treated with or without indicated concentration of RSL-3 in the presence or absence of various inhibitors. The cell viability was assessed using the Cell Counting Kit-8 according to the manufacturer’s procedure. (**A**) Time course and concentration dependency of RSL3-induced cell death. (**B**) Effect of ACSL4 knockdown on RSL3-induced cell death. (**C**,**D**) Effects of 1 µM ferrostatin-1 (Fer-1), 10 µM necrostatin-1 (Nec-1), 10 µM DPQ, or 20 µM zVAD, on HK-2 cell death induced by 1 µM RSL3 (C) or 20 µM FIN56 (D). Data represent the mean ± SD. **, *P*<0.01.

Several lines of evidence indicate that *Acsl4* is a proferroptotic gene with maintenance of oxidisable PUFA-containing PLs, such as C20:4- and/or C22:4-containing PEs [8,10]. To analyse the role of ACSL4 in ferroptosis in HK-2 cells, we used RSL3, a glutathione peroxidase 4 (GPx4) inhibitor, to induce ferroptotic cell death. As depicted in Figure 2A, RSL3 induced ferroptotic cell death in a time- and dose-dependent manner. Based on these results, the cell viability of HK-2 cells against RSL3 was evaluated in subsequent experiments 24 h after RSL3 treatment. Transfection of siRNA against ACSL4 partially, but significantly, improved RSL3-induced cell death compared with that of control siRNA (Figure 2B). Although the actual mechanisms for the partial inhibition of RSL3-induced cell death by ACSL4 knockdown is unclear, it is likely that the insufficient effects of ACSL4 knockdown on the PUFA composition of PLs are involved (Figure 1D–G). To elucidate the mechanisms of RSL3-induced HK-2 cell death, we further examined whether other cell death pathways, other than ferroptotic cell death, are also involved in RSL3-induced cell death in these cells. As shown in Figure 2C, treatment of HK-2 cells (control siRNA-transfected cells) with ferrostatin-1, a well-known ferroptosis inhibitor, strongly suppressed RSL3-induced cell death, suggesting that RSL3-induced cell death is mediated by ferroptosis. Unexpectedly, the inhibitor studies also revealed that treatment with necrostatin-1, an inhibitor of receptor-interacting protein kinase 1/3 (RIPK)1/3 signalling which executes necroptotic cell death, strongly attenuated RSL3-induced cell death. In contrast with these inhibitors, treatment of HK-2 cells with the pan-caspase inhibitor zVAD (apoptosis inhibitor) or the PARP-1 inhibitor DPQ (parthanatos inhibitor) failed to suppress RSL3-induced cell death. Similar results were also observed in RSL3-treated ACSL4 knockdown HK-2 cells (Figure 2C). Further, we also examined whether ferroptotic cell death induced by another type of ferroptotic inducer FIN56 [15] was also affected by ACSL4 knockdown or cell death inhibitors. When control or ACSL4 knockdown HK-2 cells were incubated with FIN56 for 48 h, FIN56 induced cell death in approximately 60% of control HK-2 cells whereas failed to induce the cell death in ACSL4 knockdown HK-2 cells (Figure 2D). In addition, we also found that treatment with ferrostatin-1 or necrostatin-1, but not DPQ or zVAD, also suppressed HK-2 cell death induced by FIN56.

### Effect of ACSL4 knockdown on the HK-2 cell death induced by cadmium and arsenite

**Figure 3:**
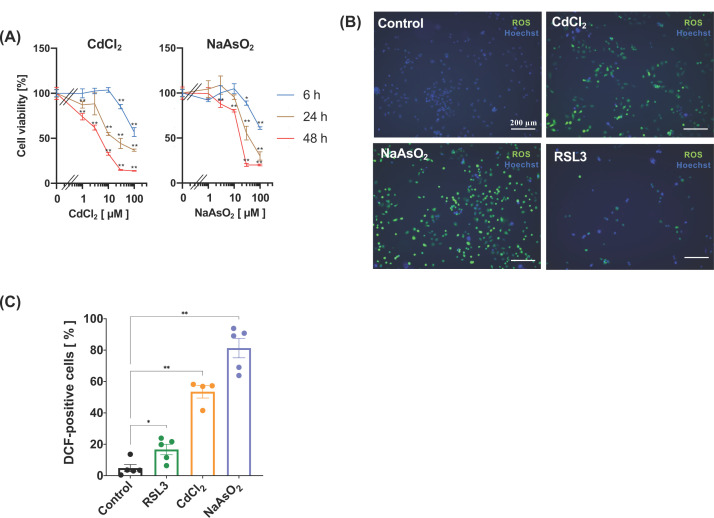
Treatment of HK-2 cells with cadmium chloride or sodium arsenite induces ROS generation (**A**) HK-2 cells grown in a 96-well plate were treated with or without indicated concentration of CdCl_2_ (*left panel*) or NaAsO_2_ (*right panel*). Cell viability at the indicated time was assessed by the Cell Counting Kit-8. Data represent the mean ± SD. **, *P*<0.01 (compared with the untreated cells). (**B**,**C**) The HK-2 cells grown in a 35-mm glass-bottom dish were treated with 1 µM RSL3, 30 µM CdCl_2_, or 30 µM NaAsO_2_ for 6 h. The ROS generated in each treatment was assessed according to the procedure detailed in the ‘Materials and methods’ section. (B) Fluorescent images of ROS generated cells (scale bar = 200 µm). (C) Quantitation of results in (B). The average percentage of ROS-positive cells in each field. Data represent the mean ± SD. *, *P*<0.05; **, *P*<0.01 (compared with the untreated cells).

We then examined whether the loss of ACSL4 protein also suppresses the toxic effects of nephrotoxic metals, such as cadmium chloride (CdCl_2_) and sodium arsenite (NaAsO_2_). As shown in Figure 3A, treatment of HK-2 cells with CdCl_2_ or NaAsO_2_ induced cell death in a time- and dose-dependent manner. To examine whether these metals up-regulate the levels of intracellular ROS, we evaluated the intracellular ROS levels after toxic metal treatments using the ROS indicator DCFH-DA. When HK-2 cells were treated with CdCl_2_, NaAsO_2_, or RSL3 for 6 h, the number of ROS-positive cells was significantly increased compared with that in untreated cells (Figure 3B,C). These results suggest that treatment of HK-2 cells with these metals generated intracellular ROS simultaneously with cellular damage.

**Figure 4:**
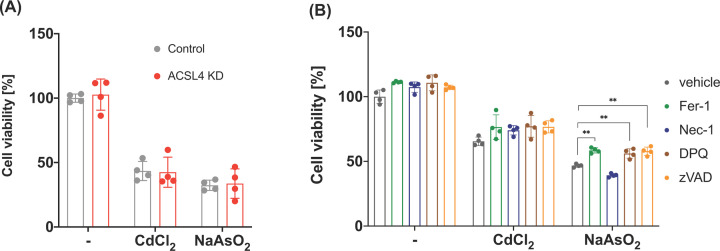
Characterisation of HK-2 cell death elicited by cadmium chloride or sodium arsenite (**A**) HK-2 cells grown in a 96-well plate were transiently transfected with ACSL4 or control siRNA (10 nM). Two days after transfection, the HK-2 cells were treated with 30 µM CdCl_2_ or 30 µM NaAsO_2_ for 24 h, and then, the cell viabilities were assessed. (**B**) The HK-2 cells cultured in a 96-well plate were treated with or without CdCl_2_ or NaAsO_2_ for 24 h and then, the cell viabilities were assessed. Data represent the mean ± SD. **, *P*<0.01.

Previous reports have revealed that intracellular ROS, which oxidises PUFA-containing PLs, participates in ferroptotic cell death induced by RSL3 [10]. In addition, the knockdown of ACSL4 in HK-2 cells significantly attenuated the RSL3-induced cell death (Figure 2B). Therefore, we speculated whether ACSL4 knockdown also affects cytotoxicity induced by CdCl_2_ or NaAsO_2_. As shown in Figure 4A, knockdown of ACSL4 failed to suppress CdCl_2_- or NaAsO_2_-induced HK-2 cytotoxicity. These results suggest that exposure of HK-2 cells to CdCl_2_ or NaAsO_2_ increases ROS, but it causes cell death independent of ACSL4.

We further examined the effects of various programmed cell death inhibitors on CdCl_2_- or NaAsO_2_-induced cell death. As shown in Figure 4B, the programmed cell death inhibitors used in the present study failed to improve CdCl_2_-induced cell death under these conditions. In contrast, treatment of HK-2 cells with ferrostatin-1, DPQ, or zVAD partially improved NaAsO_2_-induced cell death. These results show that multiple types of programmed cell death pathways are involved in NaAsO_2_-induced cell death.

## Discussion

The fatty acid composition of PLs is controlled by the deacylation and reacylation of PLs [22,23], and several enzymes, including ACSLs, are involved in a sequence of reactions. Interestingly, the levels of C20:4-containing PLs in adipocytes isolated from high-fat diet-fed adipocyte-specific *Acsl4* KO mice were significantly decreased compared with those in the control mice [6]. In contrast, the levels of C20:4-containing PLs in Acsl4 knockdown rat fibroblasts were only marginally decreased compared with those in control siRNA-transfected rat fibroblastic 3Y1 cells [18]. These results suggest that the contribution of PL remodelling enzymes in the biosynthesis of PUFA-containing PLs is different among cell types.

To extend our knowledge about the functions of ACSL4, we first investigated the effect of ACSL4 knockdown on the fatty acid content of PLs in human proximal tubule epithelial HK-2 cells. As shown in Figure 1B, knockdown of ACSL4 in HK-2 cells significantly reduced the content of the broad-spectrum PUFA-derived acyl-CoAs, except for C18:2-CoA. These results are consistent with the substrate selectivity of ACSL4 enzyme assessed by recombinant ACSL4 enzyme [7], and suggest that ACSL4 prefers C20 and/or longer PUFAs as substrates. In contrast with the levels of PUFA-derived acyl CoA, those of some saturated or monounsaturated fatty acid-derived acyl-CoA were increased in ACSL4 knockdown cells. We speculate that the contrasting effects of ACSL4 knockdown may be to compensate the reduced levels of PUFA-derived long-chain acyl-CoA by saturated and/or monounsaturated acyl-CoA species to maintain the cellular total acyl-CoA levels.

Intriguingly, it is noteworthy that although a broad spectrum of PUFA-derived acyl-CoAs declined remarkably with the knockdown of ACSL4, the levels of PLs containing PUFA, except for those containing C22:6 fatty acids, hardly decreased following the knockdown of ACSL4 expression (Figure 1D–G). These results are inconsistent with previous proposals, in which lack of ACSL4 enzyme by Acsl4 knockout dramatically decreased the levels of a broad range of PUFA-containing PLs in fibroblasts [8], adipocytes [6], and macrophages [7]. The reason that ACSL4 knockdown failed to affect the levels of PUFA-containing PLs other than C22:6-containing PLs may be explained by the contribution of other enzymes involved in PL remodelling reactions in HK-2 cells. As mentioned above, the maintenance of fatty acid composition of PLs involves the following sequential reactions: (1) deacylation of fatty acids by phospholipases A_1/2_, (2) activation of free fatty acids by ACSLs, and (3) reacylation of lysophospholipids by lysophospholipid acyltransferases. Although the actual mechanisms involved in the biosynthesis of PUFA-containing PLs in HK-2 cells are currently unknown, we speculate that the deacylation/reacylation reaction, rather than the acyl-CoA synthetase reaction, is the rate-limiting step for biosynthesis of PUFA-containing PLs in these cells and that this step delays the occurrence of the decrease in PUFA-containing PLs compared with that in PUFA-CoA. In addition, it is of interest why only C22:6 incorporation into PE and PC were reduced in the ACSL4 knockdown cells even though other PUFA-derived acyl-CoA levels, such as C20:4-/C20:5-derived acyl-CoA, were reduced. To selectively reduce the levels of C22:6-containing PLs in ACSL4 knockdown HK-2 cells, the phospholipase A_2_ reaction is likely to be a key reaction. Other possibilities include attenuation of the C22:6-CoA specific lysophospholipid acyltransferase reaction. Indeed, Shimizu and co-workers have shown that several lysophospholipid acyltransferase enzymes catalyse C22:6 as a substrate, and these enzymes contribute to the maintenance of C22:6-containing PLs [24–26]. Identification of the enzymes involved in the biosynthesis of C22:6-containing PLs, such as phospholipase A_2_ and lysophospholipid acyltransferase, in HK-2 cells may resolve why the levels of C22:6-containing PLs were selectively affected by ACSL4 knockdown in HK-2 cells.

ACSL4 is one of the key enzymes involved in PUFA metabolism and plays roles in various pathological and physiological events such as neuronal development [27,28], steroidogenesis [29], and cancer exacerbation [30,31]. More recently, it was found that the oxidation of specific PE species, such as PE (18:0/20:4) and PE (18:0/22:4), generated via the ACSL4-mediated remodelling pathway, is an important reaction for the execution of ferroptosis [8,10]. Ferroptosis is a non-apoptotic form of programmed cell death characterised by the iron-dependent accumulation of lipid peroxides [9] and is known to contribute to various diseases, including renal diseases [15]. Up-regulation of ACSL4 expression was found in patients with acute kidney tubular injury, and ferroptosis plays an important role in the progression of renal diseases [16,17]. However, the role of ACSL4 in ferroptotic cell death in renal cells is not fully understood. In this study, we found that the PUFA-containing PLs, other than C20:4- and C22:6-containing PE species, may also be involved in RSL3-induced ferroptotic cell death in renal cells. In addition, we found that the RSL3-induced HK-2 cell death was mediated by the ferroptotic and necroptotic pathways. Importantly, this observation was also observed in the case of another type of ferroptosis inducer FIN56, which promotes GPx4 degradation and antioxidant coenzyme Q10 depletion to induce ferroptosis [32,33]. Thus, renal cell death induced by ferroptosis inducers seems to be mediated via both ferroptotic and necroptotic signalling pathways.

Necroptosis is a programmed cell death process, which is executed via the activation of RIPK3/MLKL (mixed lineage kinase domain-like) pathway, accompanied by cell membrane rupture and leakage of the intracellular contents [34], and is distinct from ferroptosis. Previous studies have indicated that ferroptosis and necroptosis occur simultaneously in neuronal cell death caused by experimental intracerebral haemorrhage [35] or melanoma cell death by inhibition of mitochondrial complex I [36]. In addition, it is also reported that ferroptosis compensates for necroptosis (and *vice versa*) when either ferroptosis or necroptosis is compromised [17]. Our findings are distinct from these reports, because both ferrostatin-1 and necrostatin-1 completely cancel out RSL3/FIN56-induced HK-2 cell death. Since these two ferroptosis inducers are known to accumulate lipid ROS in the cell membranes, ferroptotic/necroptotic HK-2 cell death is likely to be triggered downstream of ROS production. Although further analyses are needed to identify the actual mechanisms for this cell death pathway observed in the present study, we speculate that activation of the RIPK3 pathway lies downstream to lipid ROS generation. Therefore, it is of interest whether lipid ROS can activate the RIPK3 pathway following the treatment of the HK-2 cells with RSL3 or FIN56.

Exposure of cells to hazardous metals, such as cadmium and arsenic, leads to cell death, the cytotoxicity of which is mediated by oxidative stress. Because ferroptosis is mediated by lipid ROS and knockdown of ACSL4 attenuates RSL3-induced HK-2 cell death, we hypothesised that the hazardous metal-induced cell death was also suppressed by the knockdown of ACSL4. As shown in Figure 3B,C, treatment of HK-2 cells with cadmium chloride or sodium arsenite significantly increased the number of ROS-accumulated cells compared with the untreated group. Under these conditions, knockdown of ACSL4 failed to attenuate both the cadmium chloride- or sodium arsenite-induced HK-2 cell death, suggesting that the signalling pathway involving ACSL4 is not the main pathway for these hazardous metal-induced HK-2 cell death. In addition, cell death induced by cadmium chloride was insensitive to the treatment with inhibitors of programmed cell death, whereas that of sodium arsenite was partially attenuated by treating with inhibitors of ferroptosis, parthanatos, and apoptosis. These results are inconsistent with previous reports that the HK-2 cell death induced by cadmium chloride is mediated by apoptosis [37] and pyroptosis [38]. These discrepancies may be due to differences in the conditions where the dose of cadmium chloride used in previous reports was low compared with our study. In contrast, arsenic-induced HK-2 cell death has been shown to mediate the complex programmed cell death pathway, including apoptosis and autophagy [39–41]. Consistent with these reports, treatment of the HK-2 cells with an inhibitor of apoptosis partially restored sodium arsenite-induced cell death. In addition, sodium arsenite-induced HK-2 cell death was partially attenuated by treating with DPQ, suggesting the involvement of parthanatos. Knockdown of ACSL4 failed to attenuate sodium arsenite-induced HK-2 cell death due to partial effects on PUFA-containing PLs, and treatment with ferrostatin-1 partially restored HK-2 cell death. Thus, the mechanisms underlying arsenic-induced HK-2 cell death are complicated due to the involvement of multiple programmed cell death pathways.

To summarise the results thus far, the mechanisms for the maintenance of PUFA-containing PLs vary between cell types, and we found that the levels of C22:6 containing PLs were influenced by the knockdown of ACSL4 in HK-2 cells. Identifying the mechanisms underlying the selective reduction in the levels of C22:6-containing PLs observed in HK-2 cells may provide new insights into understanding the remodelling pathway for PUFA-containing PLs. Additionally, HK-2 cell death induced by ferroptosis inducers was, at least in part, mediated by ACSL4 and the necroptosis-like pathway. Identifying the signalling pathways involved in programmed cell death in the renal cells might help treat several kidney diseases.

## Supplementary Material

Supplementary Figure S1 and Table S1

## Data Availability

All data are included within the main manuscript or its supplementary files.

## Abbreviations

AA: arachidonic acid
ACSL4: acyl-CoA synthetase long-chain family member 4
DCFH-DA: 2′,7′-dichlorodihydrofluorescein diacetate
FCS: foetal calf serum
GPx4: glutathione peroxidase 4
HK-2: human normal kidney proximal tubule epithelial
LC-MS/MS: liquid chromatography–tandem mass spectrometry
MRM: multiple-reaction monitoring
PC: phosphatidylcholine
PE: phosphatidylethanolamine
PL: phospholipid
PUFA: polyunsaturated fatty acid
RIPK3: receptor-interacting protein kinase 3
ROS: reactive oxygen species
siRNA: small interfering RNA
