# Supplementary material for: Role of ACSL4 in the chemical-induced cell death in human proximal tubule epithelial HK-2 cells
Source: Biosci Rep. 2022 Feb 9;42(2):BSR20212433. doi: 10.1042/BSR20212433 (PMC8829018; doi:10.1042/BSR20212433)
Supplement: Supplementary Figure S1 and Table S1 [file BSR-2021-2433_supp.pdf]

Supplement figure S1

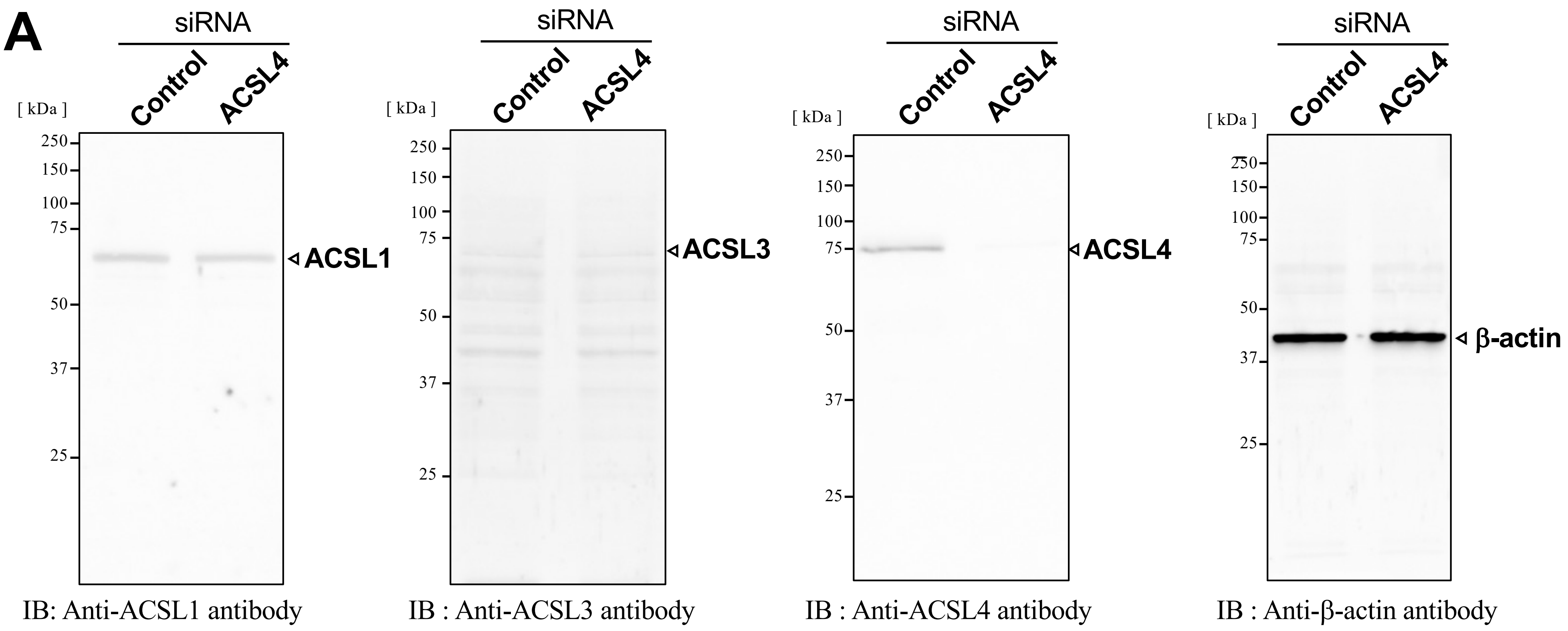

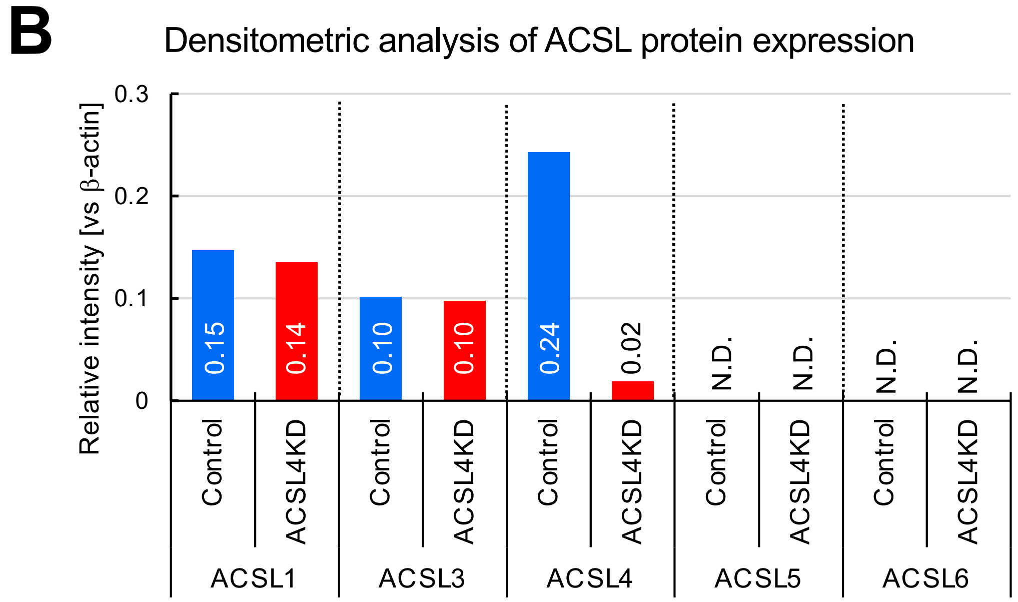

Figure legend

Figure S1. Effects of ACSL4 knockdown on ACSL protein expression. The HK-2 cells were transiently transfected with ACSL4 or control siRNA (10 nM). Two days after transfection, the HK-2 cells were subjected to immunoblotting (*A*) and densitometric analysis of ACSL protein expression (*B*). IB, immunoblotting.

Supplemental Table 1: MRMs used to detect phospholipids

Phospholipid class	Analyte	Q1 ion	Q1	Q3
PC	PC(16:0/16:0)	$[M + HCOO]^-$	778.4	255.2
	PC(16:0/16:1)	$[M + HCOO]^-$	776.4	253.2
	PC(16:0/18:0)	$[M + HCOO]^-$	806.4	283.2
	PC(16:0/18:1)	$[M + HCOO]^-$	804.4	281.2
	PC(16:0/18:2)	$[M + HCOO]^-$	802.4	279.2
	PC(16:0/20:4)	$[M + HCOO]^-$	826.4	303.2
	PC(16:0/20:5)	$[M + HCOO]^-$	824.4	301.2
	PC(16:0/22:4)	$[M + HCOO]^-$	854.4	331.2
	PC(16:0/22:5)	$[M + HCOO]^-$	852.4	329.2
	PC(16:0/22:6)	$[M + HCOO]^-$	850.4	327.2
	PC(18:0/18:0)	$[M + HCOO]^-$	834.4	283.2
	PC(18:0/18:1)	$[M + HCOO]^-$	832.4	281.2
	PC(18:0/18:2)	$[M + HCOO]^-$	830.4	279.2
	PC(18:0/20:4)	$[M + HCOO]^-$	854.4	303.2
	PC(18:0/20:5)	$[M + HCOO]^-$	852.4	301.2
	PC(18:0/22:4)	$[M + HCOO]^-$	882.4	331.2
	PC(18:0/22:5)	$[M + HCOO]^-$	880.4	329.2
	PC(18:0/22:6)	$[M + HCOO]^-$	878.4	327.2
	PC(18:1/18:1)	$[M + HCOO]^-$	830.4	281.2
	PC(18:1/18:2)	$[M + HCOO]^-$	828.4	279.2
	PC(18:1/20:4)	$[M + HCOO]^-$	852.4	303.2
	PC(18:1/20:5)	$[M + HCOO]^-$	850.4	301.2
	PC(18:1/22:4)	$[M + HCOO]^-$	880.4	331.2
	PC(18:1/22:5)	$[M + HCOO]^-$	878.4	329.2
	PC(18:1/22:6)	$[M + HCOO]^-$	876.4	327.2
	PC(16:0/16:0) alkyl	$[M + HCOO]^-$	764.4	255.2
	PC(16:0/16:1) alkyl	$[M + HCOO]^-$	762.4	253.2
	PC(16:0/18:0) alkyl	$[M + HCOO]^-$	792.4	283.2
	PC(16:0/18:1) alkyl	$[M + HCOO]^-$	790.4	281.2
	PC(16:0/18:2) alkyl	$[M + HCOO]^-$	788.4	279.2
	PC(16:0/20:4) alkyl	$[M + HCOO]^-$	812.4	303.2
	PC(16:0/20:5) alkyl	$[M + HCOO]^-$	810.4	301.2
	PC(16:0/22:4) alkyl	$[M + HCOO]^-$	840.4	331.2
	PC(16:0/22:5) alkyl	$[M + HCOO]^-$	838.4	329.2
	PC(16:0/22:6) alkyl	$[M + HCOO]^-$	836.4	327.2
	PC(18:0/16:0) alkyl	$[M + HCOO]^-$	792.4	255.2
	PC(18:0/18:0) alkyl	$[M + HCOO]^-$	820.4	283.2
	PC(18:0/18:1) alkyl	$[M + HCOO]^-$	818.4	281.2
	PC(18:0/18:2) alkyl	$[M + HCOO]^-$	816.4	279.2
	PC(18:0/20:4) alkyl	$[M + HCOO]^-$	840.4	303.2
	PC(18:0/20:5) alkyl	$[M + HCOO]^-$	838.4	301.2
	PC(18:0/22:4) alkyl	$[M + HCOO]^-$	868.4	331.2
	PC(18:0/22:5) alkyl	$[M + HCOO]^-$	866.4	329.2
	PC(18:0/22:6) alkyl	$[M + HCOO]^-$	864.4	327.2
	PC(18:1/16:0) alkenyl	$[M + HCOO]^-$	790.4	255.2
	PC(18:1/18:0) alkenyl	$[M + HCOO]^-$	818.4	283.2
	PC(18:1/18:1) alkenyl	$[M + HCOO]^-$	816.4	281.2
	PC(18:1/18:2) alkenyl	$[M + HCOO]^-$	814.4	279.2
	PC(18:1/20:4) alkenyl	$[M + HCOO]^-$	838.4	303.2
	PC(18:1/20:5) alkenyl	$[M + HCOO]^-$	836.4	301.2
	PC(18:1/22:4) alkenyl	$[M + HCOO]^-$	866.4	331.2
	PC(18:1/22:5) alkenyl	$[M + HCOO]^-$	864.4	329.2
	PC(18:1/22:6) alkenyl	$[M + HCOO]^-$	862.4	327.2
PE	PE(16:0/16:0)	$[M - H]^-$	690.5	255.2
	PE(16:0/16:1)	$[M - H]^-$	688.5	253.2
	PE(16:0/18:0)	$[M - H]^-$	718.5	283.2
	PE(16:0/18:1)	$[M - H]^-$	716.5	281.2
	PE(16:0/18:2)	$[M - H]^-$	714.5	279.2
	PE(16:0/20:4)	$[M - H]^-$	738.5	303.2
	PE(16:0/20:5)	$[M - H]^-$	736.5	301.2
	PE(16:0/22:4)	$[M - H]^-$	766.5	331.2
	PE(16:0/22:5)	$[M - H]^-$	764.5	329.2
	PE(16:0/22:6)	$[M - H]^-$	762.5	327.2
	PE(18:0/18:0)	$[M - H]^-$	746.5	283.2
	PE(18:0/18:1)	$[M - H]^-$	744.5	281.2
	PE(18:0/18:2)	$[M - H]^-$	742.5	279.2
	PE(18:0/20:4)	$[M - H]^-$	766.5	303.2
	PE(18:0/20:5)	$[M - H]^-$	764.5	301.2
	PE(18:0/22:4)	$[M - H]^-$	794.5	331.2
	PE(18:0/22:5)	$[M - H]^-$	792.5	329.2
	PE(18:0/22:6)	$[M - H]^-$	790.5	327.2
	PE(18:1/18:1)	$[M - H]^-$	742.5	281.2
	PE(18:1/18:2)	$[M - H]^-$	740.5	279.2
	PE(18:1/20:4)	$[M - H]^-$	764.5	303.2
	PE(18:1/20:5)	$[M - H]^-$	762.5	301.2
	PE(18:1/22:4)	$[M - H]^-$	792.5	331.2
	PE(18:1/22:5)	$[M - H]^-$	790.5	329.2
	PE(18:1/22:6)	$[M - H]^-$	788.5	327.2

Phospholid class	Analyte	Q1 ion	Q1	Q3
PE	PE(16:0/16:0) alkyl	$[M - H]^-$	676.5	255.2
	PE(16:0/16:1) alkyl	$[M - H]^-$	674.5	253.2
	PE(16:0/18:0) alkyl	$[M - H]^-$	704.5	283.2
	PE(16:0/18:1) alkyl	$[M - H]^-$	702.5	281.2
	PE(16:0/18:2) alkyl	$[M - H]^-$	700.5	279.2
	PE(16:0/20:4) alkyl	$[M - H]^-$	724.5	303.2
	PE(16:0/20:5) alkyl	$[M - H]^-$	722.5	301.2
	PE(16:0/22:4) alkyl	$[M - H]^-$	752.5	331.2
	PE(16:0/22:5) alkyl	$[M - H]^-$	750.5	329.2
	PE(16:0/22:6) alkyl	$[M - H]^-$	748.5	327.2
	PE(18:0/16:0) alkyl	$[M - H]^-$	704.5	255.2
	PE(18:0/18:0) alkyl	$[M - H]^-$	732.5	283.2
	PE(18:0/18:1) alkyl	$[M - H]^-$	730.5	281.2
	PE(18:0/18:2) alkyl	$[M - H]^-$	728.5	279.2
	PE(18:0/20:4) alkyl	$[M - H]^-$	752.5	303.2
	PE(18:0/20:5) alkyl	$[M - H]^-$	750.5	301.2
	PE(18:0/22:4) alkyl	$[M - H]^-$	780.5	331.2
	PE(18:0/22:5) alkyl	$[M - H]^-$	778.5	329.2
	PE(18:0/22:6) alkyl	$[M - H]^-$	776.5	327.2
	PE(18:1/16:0) alkenyl	$[M - H]^-$	702.5	255.2
	PE(18:1/18:0) alkenyl	$[M - H]^-$	730.5	283.2
	PE(18:1/18:1) alkenyl	$[M - H]^-$	728.5	281.2
	PE(18:1/18:2) alkenyl	$[M - H]^-$	726.5	279.2
	PE(18:1/20:4) alkenyl	$[M - H]^-$	750.5	303.2
	PE(18:1/20:5) alkenyl	$[M - H]^-$	748.5	301.2
	PE(18:1/22:4) alkenyl	$[M - H]^-$	778.5	331.2
	PE(18:1/22:5) alkenyl	$[M - H]^-$	776.5	329.2
	PE(18:1/22:6) alkenyl	$[M - H]^-$	774.5	327.2
PI	PI(16:0/18:0)	$[M - H]^-$	837.7	283.2
	PI(16:0/18:1)	$[M - H]^-$	835.7	281.2
	PI(16:0/18:2)	$[M - H]^-$	833.7	279.2
	PI(16:0/20:4)	$[M - H]^-$	857.7	303.2
	PI(16:0/20:5)	$[M - H]^-$	855.7	301.2
	PI(16:0/22:4)	$[M - H]^-$	835.7	331.2
	PI(16:0/22:5)	$[M - H]^-$	833.7	329.2
	PI(18:0/18:0)	$[M - H]^-$	865.7	283.2
	PI(18:0/18:1)	$[M - H]^-$	863.7	281.2
	PI(18:0/18:2)	$[M - H]^-$	861.7	279.2
	PI(18:0/20:4)	$[M - H]^-$	885.7	303.2
	PI(18:0/20:5)	$[M - H]^-$	883.7	301.2
	PI(18:0/22:4)	$[M - H]^-$	863.7	331.2
	PI(18:0/22:5)	$[M - H]^-$	861.7	329.2
	PI(18:1/18:1)	$[M - H]^-$	861.7	281.2
	PI(18:1/18:2)	$[M - H]^-$	859.7	279.2
	PI(18:1/20:4)	$[M - H]^-$	883.7	303.2
	PI(18:1/20:5)	$[M - H]^-$	881.7	301.2
	PI(18:1/22:4)	$[M - H]^-$	861.7	331.2
	PI(18:1/22:5)	$[M - H]^-$	859.7	329.2
PG	PG(14:0/14:0)	$[M - H]^-$	665.5	227.2
	PG(16:0/16:0)	$[M - H]^-$	721.5	255.2
	PG(16:0/16:1)	$[M - H]^-$	719.5	253.2
	PG(16:0/18:0)	$[M - H]^-$	749.5	283.2
	PG(16:0/18:1)	$[M - H]^-$	747.5	281.2
	PG(16:0/18:2)	$[M - H]^-$	745.5	279.2
	PG(16:0/20:4)	$[M - H]^-$	769.5	303.2
	PG(18:0/18:0)	$[M - H]^-$	777.5	283.2
	PG(18:0/18:1)	$[M - H]^-$	775.5	281.2
	PG(18:0/18:2)	$[M - H]^-$	773.5	279.2
	PG(18:0/20:4)	$[M - H]^-$	797.5	303.2
	PG(18:1/18:1)	$[M - H]^-$	773.5	281.2
	PG(18:1/18:2)	$[M - H]^-$	771.5	279.2
	PG(18:1/20:4)	$[M - H]^-$	795.5	303.2
PS	PS(14:0/14:0)	$[M - H]^-$	678.6	227.2
	PS(16:0/16:0)	$[M - H]^-$	734.6	255.2
	PS(16:0/16:1)	$[M - H]^-$	732.6	253.2
	PS(16:0/18:0)	$[M - H]^-$	762.6	283.2
	PS(16:0/18:1)	$[M - H]^-$	760.6	281.2
	PS(16:0/18:2)	$[M - H]^-$	758.6	279.2
	PS(16:0/20:4)	$[M - H]^-$	782.6	303.2
	PS(18:0/18:0)	$[M - H]^-$	790.6	283.2
	PS(18:0/18:1)	$[M - H]^-$	788.6	281.2
	PS(18:0/18:2)	$[M - H]^-$	786.6	279.2
	PS(18:0/20:4)	$[M - H]^-$	810.6	303.2
	PS(18:1/18:1)	$[M - H]^-$	786.6	281.2
	PS(18:1/18:2)	$[M - H]^-$	784.6	279.2
	PS(18:1/20:4)	$[M - H]^-$	808.6	303.2
Internal standard	PC(14:0/14:0) IS	$[M + HCOO]^-$	722.4	227.2
	PE(14:0/14:0) IS	$[M - H]^-$	634.5	227.2
